# Substitution of tryptophan 89 with tyrosine switches the DNA binding mode of PC4

**DOI:** 10.1038/srep08789

**Published:** 2015-03-05

**Authors:** Jinguang Huang, Yanxiang Zhao, Huaian Liu, Dan Huang, Xiankun Cheng, Wensheng Zhao, Ian A. Taylor, Junfeng Liu, You-Liang Peng

**Affiliations:** 1 State key Laboratory of Agrobiotechnology, China Agricultural University, No2 Yunamingyuanxilu, Beijing. 100193, China; 2 MOA Key Laboratory of Plant Pathology, China Agricultural University, No2 Yunamingyuanxilu, Beijing. 100193, China; 3 College of Agronomy and Plant Protection, Qingdao Agricultural University, Qingdao, Shandong. 266109, China; 4 Molecular Structure, MRC-NIMR, The Ridgeway, London, NW7 1AA, United Kingdom

## Abstract

PC4, a well-known general transcription cofactor, has multiple functions in transcription and DNA repair. Residue W89, is engaged in stacking interactions with DNA in PC4, but substituted by tyrosine in some PC4 orthologous proteins. In order to understand the consequences and reveal the molecular details of this substitution we have determined the crystal structures of the PC4 orthologue MoSub1 and a PC4 W89Y mutant in complex with DNA. In the structure of MoSub1-DNA complex, Y74 interacts directly with a single nucleotide of oligo DNA. By comparison, the equivalent residue, W89 in wild type PC4 interacts with two nucleotides and the base of the second nucleotide has distinct orientation relative to that of the first one. A hydrophobic patch around W89 that favours interaction with two nucleotides is not formed in the PC4 W89Y mutant. Therefore, the change of the surface hydrophobicity around residue 89 results in a difference between the modes of DNA interaction. These results indicate that the conserved Y74 in MoSub1 or W89 in PC4, are not only key residues in making specific interactions with DNA but also required to determine the DNA binding mode of PC4 proteins.

Sub1 (Suppressor of TFIIB) or TSP1 (transcriptional stimulatory protein) is a transcription cofactor originally isolated from the budding yeast *Saccharomyces cerevisiae*. Sub1 orthologous proteins, including the human PC4, have single-stranded DNA (ssDNA) binding, DNA unwinding activity and multiple functions in transcription and DNA repair^1^,^2^,^3^,^4^,^5^,^6^,^7^. In recent studies, Sub1 was shown to be present in the RNA polymerase II pre-initiation complexes (PICs) along with the replication protein-A (RPA)^8^, and to stimulate RNA polymerase III initiation^6^.

Nucleic acid binding is the key factor for Sub1's function in DNA-dependent processes such as transcription and DNA repair^7^. The DNA binding domains in Sub1 and PC4, the human orthologue, have been characterized using biochemical methods^3^,^4^. Structures of the DNA binding domains of PC4 as well as MoSub1 from the rice blast fungus *Magnaporthe oryzae* have been determined^9^,^10^,^11^ and more recently, structures of two other PC4 orthologues from *Lactococcus lactis* and bacteriophage T5, have also been solved^12^,^13^. In PC4, the dimeric DNA binding domain is located in a C-terminal region spanning amino acids 63–127 (PC4-CTD), and is comprised of a curved five strand anti-parallel β-sheet followed by one α-helix. The dimer is formed through interaction of the α-helix of one monomer with the convex surface of the β-sheet of the other monomer^9^,^10^. ssDNA is tightly bound to the concave β-surfaces of the PC4 homodimer, adopting a hairpin-like conformation. Several positively charged side chains (R70, R86, R100, and K101) interact with the backbone of DNA either through the formation of direct hydrogen bonds or through water-mediated interactions^10^. Together with these hydrogen bond interactions, F77 and W89 engage in stacking interactions with several DNA bases and mutation of either residue to alanine abolishes ssDNA binding^4^. F77 is conserved in all Sub1 orthologous proteins (Interpro domain IPR003173) of nine key species, while W89 is substituted by tyrosine in the proteins of six species (rice, mouse-ear cress, fruit fly, nematode, budding and fission yeast) as well as MoSub1 in *Magnaporthe* oryzae (Figure 1)^11^.

Although tryptophan/tyrosine substitution is considered a relatively conservative change^14^, some reports have demonstrated that tryptophan to tyrosine mutations can have large effects on the proteins function^15^,^16^. In order to determine the effect of tryptophan/tyrosine substitutions of PC4 orthologous proteins, we have determined the crystal structures of the DNA complexes of the PC4 W89Y mutant and MoSub1 and analysed the effect of the W89Y mutation of PC4 on DNA binding affinity. These data provide the molecular details of how the mode of DNA recognition is switched by tryptophan to tyrosine substitution in this family of transcriptional cofactors.

## Methods

### Site-directed mutagenesis

The PC4 W89Y mutant construct was prepared by Sino-US Taihe Co. The mutant allele was amplified by PCR from the plasmid pHAT2-PC4DBD with two overlapping primers containing the target mutation. After digestion with *Dpn*I, the PCR product was transformed into *Escherichia coli* JM109 competent cells, and positive clones with the mutation at the position 89 were identified by PCR and confirmed by DNA sequencing.

### Expression and purification of the wild-type or mutant proteins

Expression and purification of the N-terminal His6-tagged MoSub1 in *Escherichia coli* were performed as previously described^11^. The wild-type and mutant PC4-CTD were expressed and purified in the same way as MoSub1. Expression of the target proteins was induced by addition of 0.1 mM IPTG in *E. coli* BL21 (DE3) followed by incubation with shaking at 289 K overnight. Cells were pelleted by centrifugation and lysed via sonication. Recombinant proteins were purified using Chelating Sepharose Fast Flow Agarose, Resource Q anion-exchange and Superdex 200 10/300 GL gel-filtration chromatography (GE Healthcare) following the instructions provided by the manufacturers.

### Measurement of DNA binding affinity of proteins by isothermal titration calorimetry (ITC)

The DNA binding affinity of the wild type and W89Y mutant PC4 or wild type MoSub1 was measured by ITC. The protein (300–600 μM of monomer) was titrated into the ITC-200 (Microcal) cell containing 15–50 μM of d(T_4_GGAGGT_4_) or d(T_5_GGAGGT_5_). Solutions containing proteins and oligo DNA were prepared in the same buffer (20 mM Tris-HCl pH 8.0, 300 mM NaCl). Each experiment was performed at 298 K with a preliminary injection of 2 μL of target proteins. The stirring speed was 1000 rpm and the thermal power was recorded every 5 s. The heat of dilution, measured through injection of the protein into the same buffer assay solution, was subtracted from each titration to obtain the net reaction heat value. Each experiment was done in duplicate and thermogram analysis was performed using the Origin 7.0 package with a one-site binding model in the ORIGIN software.

### Crystallization

Initial crystals of MoSub1 or PC4 W89Y mutant were obtained by sitting drop vapour diffusion using a homemade crystallization screen (“Lesley's IC” screen or salt screen) dispensed with an Oryx4 or Oryx8 crystallisation robot (Douglas Instruments Ltd.). Briefly, 0.1 μL of each protein-DNA (oligo dT_19_G_1_) mixture (1:1–1.2) was mixed with 0.1 μL of well solution and equilibrated against a 73 μL reservoir of well solution. Crystals appeared after approximately one month in the condition of 2.2–2.4 M ammonium sulphate, 0.1 M citric acid pH 5.0 (for the MoSub1-DNA complex) or 0.8 M K_2_HPO_4_-NaH_2_PO_4_ pH 6.9 (for the PC4-W89Y mutant). The crystals were flash-cooled by immersion in LV cryoOil (MiTeGen, LLC) before being plunged into liquid nitrogen. They were then stored in liquid nitrogen until being used in X-ray diffraction experiments.

### Data collection, structure determination, and structure analysis

Diffraction data were collected at 100 K on Diamond light source beamline I02, UK for the MoSub1-DNA complex and at Shanghai Synchrotron Research Facility station BL-17U, China for the PC4 W89Y-DNA complex. The data were indexed and scaled with Xia2 (XDS) in CCP4^17^,^18^,^19^. The resulting data collection and refinement statistics are summarized in Table 1. The crystals of the MoSub1-DNA complex belong to space group P2_1_ (a = 84.2 Å, b = 58.0 Å, c = 83.8 Å, and β = 107.0°), while the crystals of the PC4 W89Y-DNA complex belong to space group P4_1_2_1_2, (a = b = 67.7 Å and c = 120.4 Å). Structures were solved by molecular replacement using Phaser^20^, with the MoSub1 apo-structure (PDB entry 4AGH) or PC4-DNA complex structure (PDB entry 2C62) stripped of waters, DNA or ligands as the search models respectively. The asymmetric unit is estimated to contain six monomers with a solvent content of 32% for the MoSub1-DNA complex or two monomers with a solvent content of 76% for the PC4 W89Y -DNA complex.

**Figure 1:**
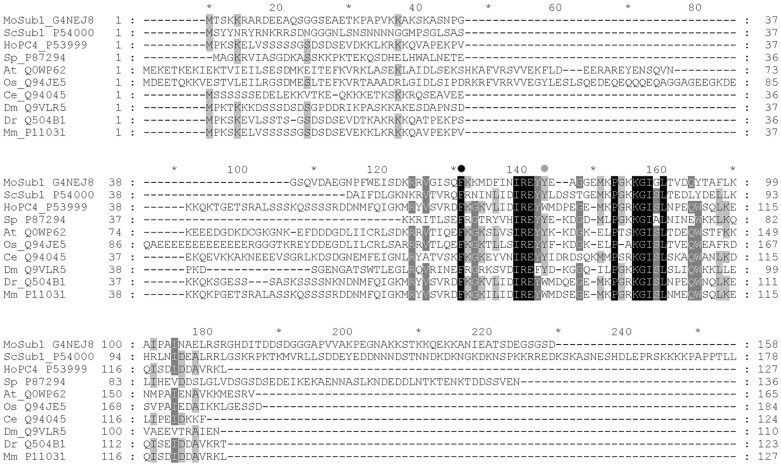
Sequence alignment of the MoSub1 (G4NEJ8) with its orthologues in *Homo sapiens* (HoPC4_P53999), *Saccharomyces cerevisiae* (ScSub1_P54000), *Schizosaccharomyces pombe* (Sp_P87294), *Arabidopsis thaliana* (At_Q0WP62), *Oryza sativa* subsp. *Japonica* (Os_Q94JE5), *Caenorhabditis elegans* (Ce_Q94045), *Drosophila melanogaster* (Dm_Q9VLR5), *Danio rerio* (Dr_Q504B1), and *Mus musculus* (Mm_P11031). The conserved F77 and substituted W89 of PC4 are indicated by black and grey dots above the sequences.

The initial models were subsequently improved by manual building in Coot^21^ and further refined using PHENIX with TLS restraints^22^,^23^. Atomic coordinates and structure factors of the MoSub1-DNA complex and PC4 W89Y-DNA complex have been deposited in the Protein Data Bank with the codes 4BHM and 4USG, respectively. Crystal parameters and data-collection statistics are listed in Table 1. Stereochemical validation of the model was performed with MolProbity^24^. The structural figures were generated with PyMOL (The PyMOL Molecular Graphics system, Version 1.3 Schrodinger, LLC) and protein-DNA contacts were analysed by LigPlot+^25^. Sequence alignment was generated with ClustalW^26^ and Boxshade (http://www.ch.embnet.org/software/BOX_form.html) was used to prepare Figure 1.

## Results

### Structural comparison of the W89Y mutant and wild type of PC4-DNA complexes

**Figure 2:**
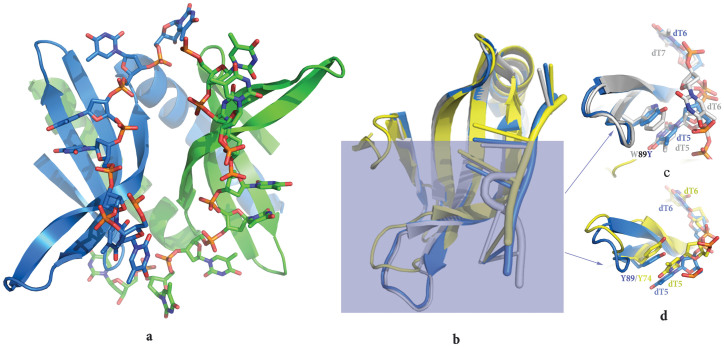
The overall structure of the PC4 mutant-ssDNA complex and the comparison of the structure of PC4 wild type or mutant DNA complexes with MoSub1 DNA-bound structure. Overall structure of the PC4 mutant-ssDNA complex (a). Chains A-B, shown in cartoon representation, are coloured in blue and green respectively. Superposition of the monomer structure of PC4 wild type (grey), PC4 mutant (blue) and MoSub1 (yellow) DNA complex (b). The region around residue 74 or 89 is highlighted to show different DNA conformations in the PC4 mutant and PC4 wild type (c), PC4 mutant and MoSub1 (d) DNA complexes.

The PC4 W89Y mutant protein was expressed in *E. coli* and purified in the same way as MoSub1^11^. In order to evaluate effects of the W89Y mutation on the configuration of DNA binding, the PC4 mutant-DNA complex was crystallized and its structure was solved at 1.97 Å resolution by molecular replacement. Data collection and model refinement statistics are presented in Table 1; representative electron density for the DNA is shown in Supplementary Figure 1. The asymmetric unit (ASU) comprises a single protein dimer and two ssDNA chains. The final crystallographic model contains residues 62–127 of both molecules of PC4 together with two ssDNA chains. Each ssDNA chain (6 or 8 nucleotides) is bound by one subunit of the PC4 mutant (Figure 2a).

**Figure 3:**
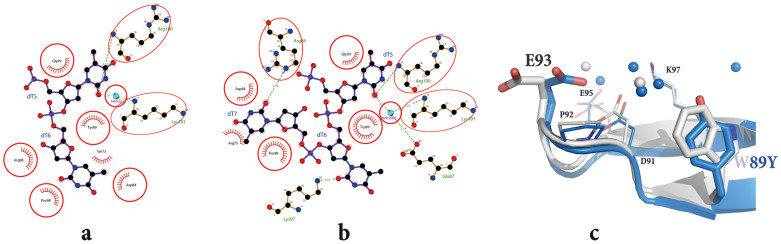
Differences between the wild type and mutant PC4 in the protein-DNA interface. Schematic illustrations (Ligplot+) of the protein-DNA contacts around residue 89 in the W89Y mutant (a) and wild type (b) of the PC4-DNA complexes. The conformation of residues around position 89 in the wild type and W89Y mutant DNA complex (c). The water molecules in this region are coloured in grey and blue for the wild type and mutant complexes, respectively.

Comparison with the structure of the DNA complex of the wild-type and that of mutant PC4, yields a RMSD between equivalent α-carbon atoms of ~0.4 Å (Figure 2b), indicating that the W89Y mutation induces little or no apparent change to the main chain of the structure of PC4. However, although the wild type and W89Y mutant of PC4 have the same conserved DNA binding motif, the DNA conformation around residue 89 in the mutant-DNA complex differs from that in the wild type complex (Figure 2b, 2c, Supplementary Figure 2a–2b). In the wild type PC4-DNA complex, residue W89 interacts with two nucleotides by hydrophobic and base-stacking interactions (Figure 2c, Supplementary Figure 2b). The base of the second nucleotide (dT6), unlike that of first nucleotide (dT5), has a vertical angle relative to side chain of W89. However, in the PC4 mutant-DNA complex residue Y89 interacts with only the dT5 nucleotide base that adopts the same conformation as in the wild-type complex (Figure 2c; Supplementary Figure 2a). As the conformation of the DNA in the binding site was modified in the W89Y mutant DNA complex compared to that of the wild type, the interactions between nucleotides and other residues around W89 in the wild type or W89Y mutant were analysed in detail (Figure 3a–3c). The interactions with the dT5 nucleotide in the two complexes are conserved and formed by four or five residues (Y/W89, G99, R100, and K101) (Figure 3a, 3b). Four other residues (D84, R86, P98, and S73), which interact with the second nucleotide (dT6) in the mutant complex, form the interactions with the third nucleotide (dT7) in the wild type protein complex, although residue S73 was replaced by R75. The second nucleotide in the wild type PC4-DNA complex interacts with K97 and W89 of PC4, and these interactions are not observed in the mutant complex (Figure 3a, 3b).

### Structural features of the DNA binding site of MoSub1

**Figure 4:**
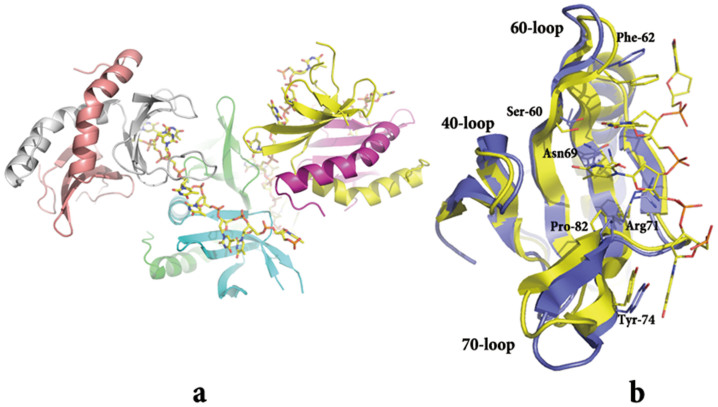
The overall structure of the MoSub1-ssDNA complex and comparison between DNA-bound and DNA-free structures of MoSub1. Overall structure of the MoSub1-DNA complex (a). Chains A-F, shown in cartoon representation, are coloured in cyan, green, yellow, magenta, brown, and grey separately. DNA is shown in stick representation. Superposition of the structures of MoSub1 DNA-free (blue) and MoSub1-DNA complex (yellow) (b).

The structure of the MoSub1-DNA complex was determined at 2.7 Å resolution by molecular replacement. Data collection and model refinement statistics are given in Table 1. The asymmetric unit (ASU) comprises six protein molecules and three ssDNA chains. The final crystallographic model contains residues 38–116 or 39–116 of all six molecules of MoSub1 together with three ssDNA chains with either 7 or 8 nucleotides visible (Figure 4a, Supplementary Figure 1). Each chain of ssDNA is bound by two monomers from two different dimers of MoSub1 (Figure 4a), which differs from the PC4-DNA complex in which PC4 binds to DNA with two monomers from the same dimer^10^. Nevertheless, the ssDNA is still bound by the same DNA-binding motif (Figure 4b) predicted by the apo- and PC4-DNA complex structures^10^,^11^. The six protein chains (A-F) form three dimers (AB, CD and EF). Each protein subunit contains a curved five stranded anti-parallel β-sheet (β1-β5) followed by one α-helix, which is a key element in the dimer interface. Superposition of Cα atoms between chains of the structure indicates a high level of structural similarity with RMSDs less than 0.7 Å and all the ssDNA molecules interacting with the chains are in nearly identical positions. Based on these results, chain F of the model was unbiasedly chosen to analyse the protein-DNA interactions.

As observed in the apo-structure, no electron density is visible for the N-terminal 1–38 and C-terminal 117–158 residues in chain F. Therefore, it is likely that these regions are disordered even after binding with DNA. Similarly, only eight nucleotides (labelled as dT1-8) of the twenty nucleotides (oligo dT_19_G_1_) are ordered and built in the final model. When chain F of MoSub1 is superposed on the DNA-free structure, the RMSD of the Cα positions is ~1.0 Å, indicating that DNA binding induces little or no changes in the structure of MoSub1. The only distinct structural difference upon DNA binding is in the region of three loops (40, 60, and 70-loop), which are close to the DNA binding surface (Figure 4b). The major interface with ssDNA (dT4 to 8) is formed by three β-strands (3–5) that are linked by the 60-loop and 70-loop and it appears that a number of residues in the interface, including F62, Y74, P82, S60, and N69, close in on the ssDNA upon interaction (Figure 4b). The interface is comprised mainly of side chain interactions from the ten residues that make direct contacts with dT5 to dT8 of ssDNA (Supplementary Figure 2c,). Most of these interactions occur between the bases of ssDNA and aromatic residues, such as Y74 and F62 that are conserved in the PC4-DNA complex. Other residues, including N69, R71, P82, and T90, make hydrophobic contacts with ssDNA through the aliphatic parts of their side chains. In addition, there are also direct hydrogen bond interactions with the DNA bases mediated by S60, K84, and Q93.

### Structural comparison of the MoSub1 and PC4-W89Y mutant DNA complexes

The structures of the DNA binding domain of MoSub1 and the PC4 W89Y mutant are highly similar. The RMSDs after superposition of Cα positions between the chain F of MoSub1 and human PC4 W89Y mutant -DNA complex is 1.5 Å (Figure 2b). Concomitantly, as predicted from the apo-structure, MoSub1 uses the same surface formed by three β-strands (3–5) to bind ssDNA as PC4^10^,^11^ and many of the residues involved in the protein-DNA interactions are conserved in the two complexes (Supplementary Figure 2a, 2c). Among them, Y74 of MoSub1 interacts with a single nucleotide in a similar way as Y89 of PC4 mutant (Figure 2d). A comparative analyses of the protein-DNA interactions around this region reveals that while W89 of PC4 stacks between two successive bases, its equivalent Y74 in MoSub1 or W89Y mutant of PC4 only stacks with one (Figure 2c–d) showing that substitution of tryptophan by tyrosine in the DNA binding sites of MoSub1 and PC4 results in a switching of the mode of DNA binding.

### DNA binding affinity of wild type, W89Y mutant of PC4 and MoSub1

To validate or structural model, the effect of W89Y substitution on the DNA binding affinity of PC4 with ssDNA was analysed by ITC (Supplementary Figure 3a–f). These data showed that PC4 binds to ssDNA oligo d(T_4_GGAGGT_4_) and d(T_5_GGAGGT_5_) relatively weakly with equilibrium dissociation constants 3.39 μM and 0.617 μM, respectively (Supplementary Figure 3a–b), consistent with a weak PC4-DNA interaction determined by electrophoretic mobility shift assays (EMSA)^3^. Additionally, the analysis of the DNA binding affinity of the PC4 W89Y mutant or MoSub1 to the same d(T_4_GGAGGT_4_) and d(T_5_GGAGGT_5_) oligonucleotides showed a similar affinity with the wild type. They also had the same trend of increasing affinity with increasing length of oligo dT (Supplementary Figure 3c–f), indicating that whilst the mode DNA binding is altered as a result of the W89Y mutation it does not significantly affect the overall binding affinity to GGAGG linked tandem dT5 or dT4 ssDNA repeat sequences.

## Discussion

Sub1 and other PC4 orthologous proteins have multiple functions in transcription and DNA repair^6^,^8^. The ssDNA binding and DNA unwinding activity of these factors are keys for their multi-function^7^.

W89, one of two key residues engaging in stacking interactions with several DNA bases in PC4^9^,^10^, is substituted with tyrosine in some PC4 orthologous proteins from fungi and plants (Figure 1). Among 20 natural amino acids, tryptophan is structurally similar to tyrosine both containing a hydrogen atom donor within an aromatic group side-chain and the mutation from tryptophan to tyrosine has been suggested to be relatively conserved^14^. Therefore it might be anticipated that substitution of tryptophan with tyrosine in PC4 orthologous proteins would not have a large effect on their function. Indeed, our data show that the DNA binding affinity for dT stretches of wild type and W89Y mutant are relatively similar (Supplementary Figure 3). However, even though the structures DBD of PC4 in the wild-type and mutant DNA complex are virtually identical, the DNA conformation changes remarkably after the substitution (Figure 2b, 2c). In the wild type PC4-DNA complex, residue W89 interacts with two nucleotides through both hydrophobic and a base-side chain stacking interaction. The interaction with the first nucleotide (dT5) is through stacking and this interaction is also observed in the W89Y mutant and with Y74 in the MoSUB1 complex. In contrast interaction with the base of the second nucleotide (dT6) that packs perpendicularly to the aromatic ring of W89 (Figure 2c) is not observed in either the W89Y mutant or the MoSub1 complex. These results revealed that the change in DNA conformation between MoSub1 or PC4 W89Y mutant and PC4 DNA complexes is caused by the tryptophan/tyrosine substitution and tryptophan can bind to two bases of DNA whilst tyrosine can only make the stacking interaction with a single base of the DNA.

Previous reports have also revealed that tyrosine/tryptophan substitutions can have significant effects on the function nucleic acid binding proteins^15^,^16^. In the human DNA repair enzyme alkyl-adenine DNA glycosylase (AAG) tyrosine/tryptophan substitution mutants in the base-binding site maintain the same nucleic acid binding affinity but dramatically affect the rate constants for nucleotide flipping in and out of the active site^15^. These observations together with our data suggest that tryptophan and tyrosine can make very different interactions with the flipped 1, N^6^-ethenoadenine (εA) base in AAG and alter the kinetics of εA flipping and excision.

Both tyrosine and tryptophan are so similar in terms of side chain volume and capacity to stack against DNA bases. Therefore, one possibility for the observed functional consequences of substitution could result from the phenolic oxygen atom of tyrosine disfavouring any alternative hydrophobic interactions that a tryptophan can make with nucleobases. In support of this notion, in wild type PC4, W89 and surrounding residues form an apolar patch that accommodates the second of the two nucleobases “edge on” through hydrophobic interactions. However, substitution of tryptophan with tyrosine in the mutant disrupts the hydrophobic patch and also results in reorientation of the nearby residue, E93 (Figure 3c). As a result, the second nucleobase does not move close to Y89 to form the interactions observed in the wild type complex. Therefore, although Y74 of MoSub1 or W89Y PC4 mutant maintain the aromatic nature and still undergo stacking interaction with one DNA base, it is possible that the presence of the tyrosine phenolic oxygen is unfavourable for interaction with a second nucleotide. In this way, W89 of PC4 or Y74 of MoSub1 direct the mode of the DNA-protein interaction and the two conformations of DNA observed in these DNA complexes might represent differences in strategy used by PC4 and Sub1 to either unwind DNA or bind to the unwound DNA. Elucidation of the relationship between these respective DNA binding modes and their impact on function will be an interesting topic for future research.

## Author Contributions

I.T., J.L. and Y.P. designed experiments. J.H., Y.Z., H.L., D.H. and X.C. performed the experiments. J.H., Y.Z. and W.Z. analysed the results. I.T., J.L. and Y.P. wrote the paper. All of the authors offered a critical review of the paper.

## Supplementary Material

Supplementary Information Supplementary Information

## Figures and Tables

**Table 1:** Data collection and refinement statistics

	MoSub1-DNA	PC4(W89Y)-DNA
Wavelength (Å)	0.9715	0.9795
Resolution range (Å)	30.0–2.7 (2.85–2.70)	47.8–1.97 (2.04–1.97)
Space group	P 2_1_	P 4_1_ 2_1_ 2
Unit cell (Å)	84.2 58.0 83.8 90 107.0 90	67.7 67.7 120.4 90 90 90
Total reflections	58926	40774
Unique reflections	20278	20387
Multiplicity	2.9 (2.8)	2.0 (2.0)
Completeness (%)	94.33 (98.54)	99.99 (99.90)
Mean I/sigma (I)	6.33 (2.15)	24.29 (6.79)
Wilson B-factor (Å^2^)	43.28	24.09
R-merge	0.071 (0.315)	0.01405 (0.09722)
CC(1/2)	0.995(0.844)	1 (0.969)
R-factor	0.1839 (0.2945)	0.1853 (0.2184)
R-free	0.2434 (0.3789)	0.2120 (0.2519)
Number of atoms	4268	1494
Macromolecules	4153	1366
Ligands (SO_4_)	25	/
Water	137	128
Protein residues	486	146
RMS (bonds) (Å)	0.008	0.009
RMS (angles)(°)	1.26	1.19
Ramachandran favoured (%)	96	100
Ramachandran outliers (%)	0	0
Clash score	12.98	7.21
Average B-factor (Å^2^)	54.90	36.20
Macromolecules (Å^2^)	54.70	35.60
Solvent (Å^2^)	41.20	42.60

Highest resolution shell is shown in parenthesis.

CC(1/2) = percentage of correlation between intensities from random half-datasets.
