# Supplementary material for: Substitution of tryptophan 89 with tyrosine switches the DNA binding mode of PC4
Source: Sci Rep. 2015 Mar 5;5:8789. doi: 10.1038/srep08789 (PMC4350104; doi:10.1038/srep08789)
Supplement: Supplementary Information [file srep08789-s1.pdf]

Substitution of tryptophan 89 with tyrosine switches the DNA binding mode of PC4

Jinguang Huang[abc1], Yanxiang Zhao[b1], Huaian Liu[b], Dan Huang[b], Xiankun Cheng[b], Wensheng Zhao[ab], Ian A. Taylor[d], Junfeng liu[b*] and You-Liang Peng[ab]

[a]State key Laboratory of Agrobiotechnology, [b]MOA Key Laboratory of Plant Pathology, China Agricultural University, No2 Yunamingyuanxilu, Beijing, 100193, China; [c]College of Agronomy and Plant Protection, Qingdao Agricultural University, Qingdao, Shandong, 266109, China, and [d]Molecular Structure, MRC-NIMR, The Ridgeway, London, NW7 1AA, United Kingdom.

[1] These authors contributed equally to this work.

Correspondence Email: jliu@cau.edu.cn

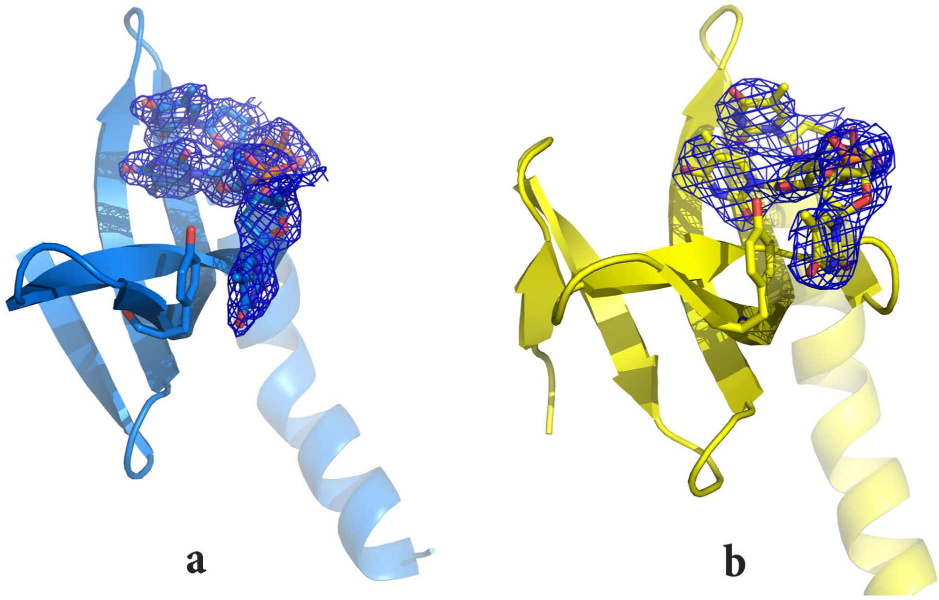

Supplementary Figure 1. 2Fo-Fc electron density maps (blue mesh) contoured at 1.5σ of the DNA around Tyr89 within PC4 W89-DNA complex (a) and Tyr74 in the MoSub1-DNA complex (b).

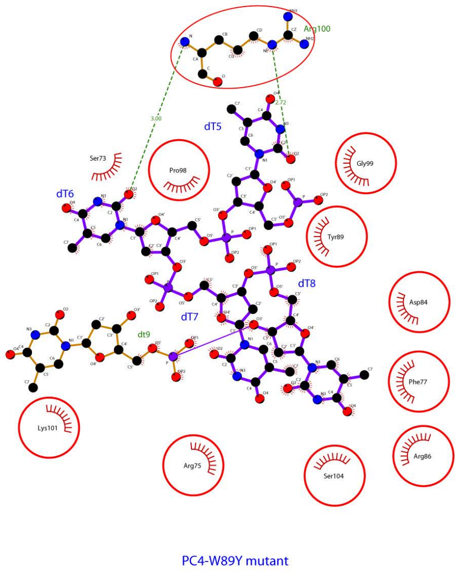

a

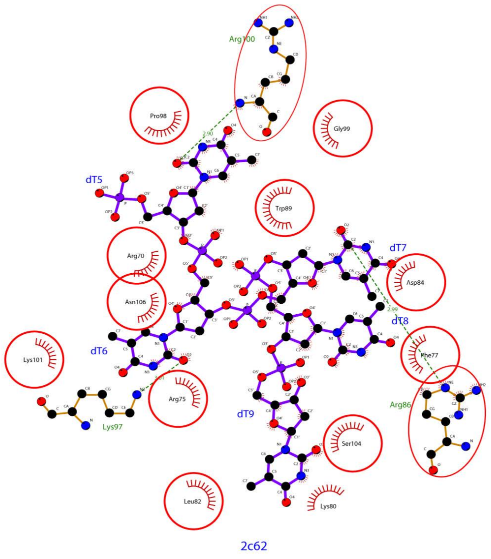

b

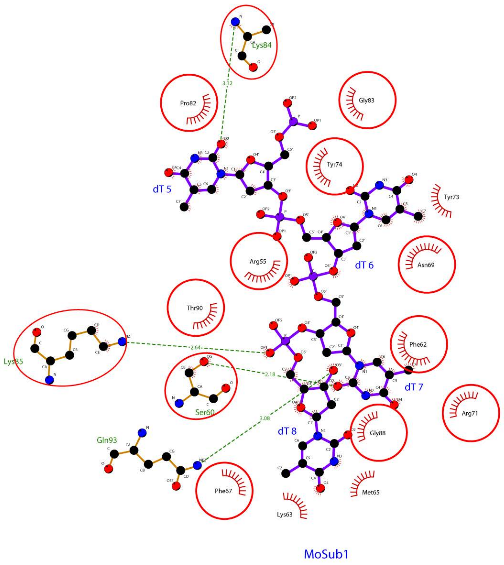

c

Supplementary Figure 2. The schematic illustrations (Ligplot+) of the protein-DNA contacts in the PC4 W89Y mutant-DNA complex (a), PC4-DNA complex(PDB 2C62) (b), and the MoSub1-DNA complex (c).

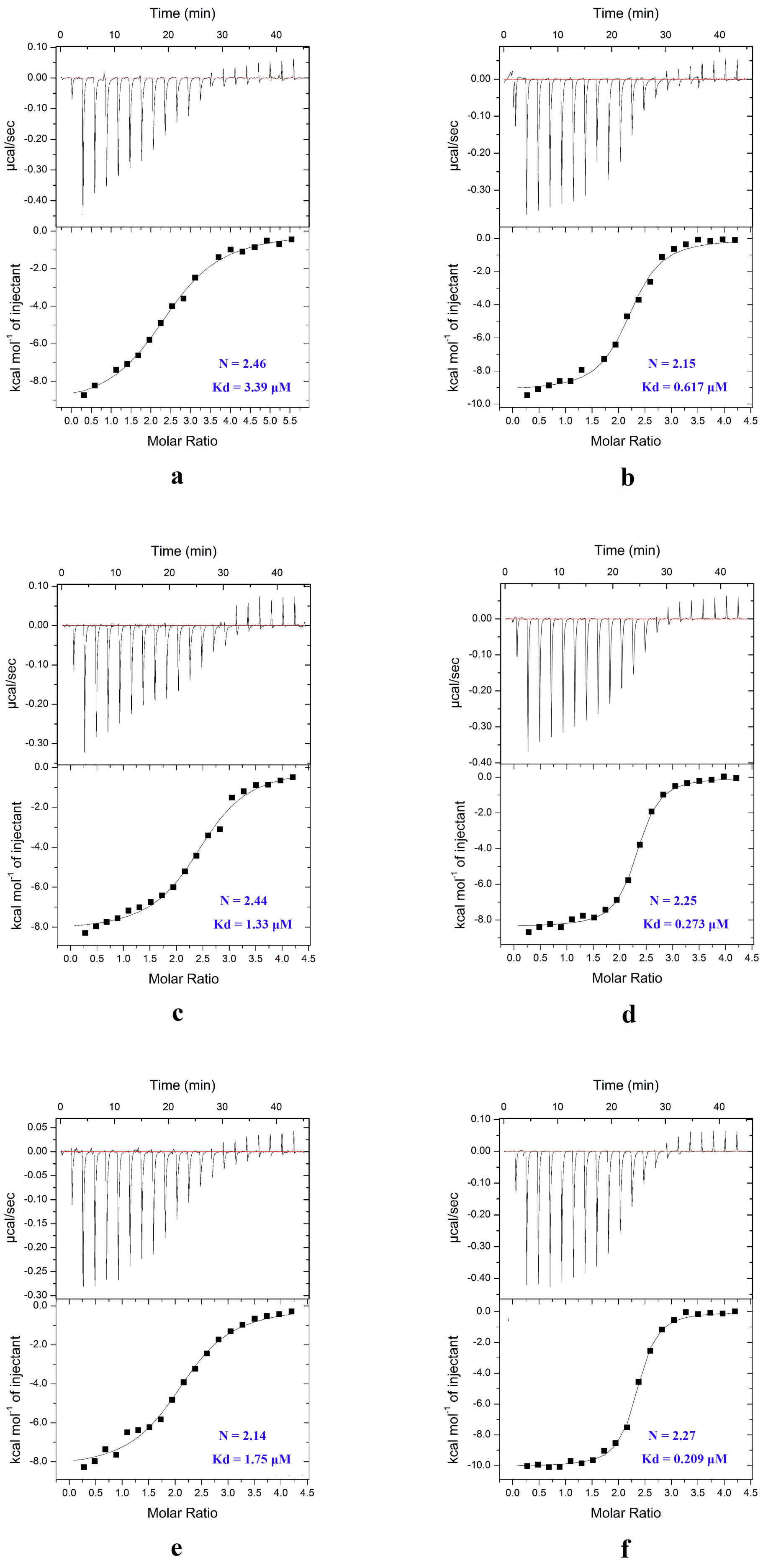

Supplementary Figure 3. Representative Isothermal Titration calorimetry (ITC) curves of wild type (a, b) or W89Y mutant (c, d) of PC4, and MoSub1 (e, f), binding with d(T4GGAGGT4) or d(T5GGAGGT5). Upper panels show the raw data, bottom panels show the integrated data. Experimental data were fitted using the 'identical and independent sites' binding models in the Origin Software (Origin Lab).
